# Construction and Evaluation of Small-Diameter Bioartificial Arteries Based on a Combined-Mold Technology

**DOI:** 10.3390/polym14153089

**Published:** 2022-07-29

**Authors:** Weijie Jiao, Chen Liu, Jingxin Shan, Zhiyuan Kong, Xiaohong Wang

**Affiliations:** 1 Center of 3D Printing & Organ Manufacturing, School of Intelligent Medicine, China Medical University (CMU), Shenyang 110122, China; i897708376@163.com (W.J.); 18856152351@163.com (C.L.); shanjingxin@huh.edu.cn (J.S.); 2020121772@stu.cmu.edu.cn (Z.K.); 2 Center of Organ Manufacturing, Department of Mechanical Engineering, Tsinghua University, Beijing 100084, China

**Keywords:** small-diameter arterial vessels, adipose-derived stem cells (ASCs), combined mold, growth factors, gelatin/alginate hydrogels, polylactic–glycolic acid (PLGA)

## Abstract

Arterial stenosis or blockage is the leading cause of cardiovascular disease, and the common solution is to substitute the arteries by autologous veins or bypass the blood vessels physically. With the development of science and technology, arteries with diameter larger than 6 mm can be substituted by unbiodegradable polymers, such as polytetrafluoroethylene, clinically. Nevertheless, the construction of a small-diameter (less than 6 mm) artery with living cells has always been a thorny problem. In this study, a suit of combined mold was designed and forged for constructing small-diameter arterial vessels. Based on this combined mold, bioactive arterial vessels containing adipose-derived stem cells (ASCs) and different growth factors (GFs) were assembled together to mimic the inner and middle layers of the natural arteries. Before assembling, ASCs and GFs were loaded into a gelatin/alginate hydrogel. To enhance the mechanical property of the bilayer arterial vessels, polylactic–glycolic acid (PLGA) was applied on the surface of the bilayer vessels to form the outer third layer. The biocompatibility, morphology and mechanical property of the constructed triple-layer arterial vessels were characterized. The morphological results manifested that cells grow well in the gelatin/alginate hydrogels, and ASCs were differentiated into endothelial cells (ECs) and smooth muscle cells (SMCs), respectively. In addition, under the action of shear stress produced by the flow of the culture medium, cells in the hydrogels with high density were connected to each other, similar to the natural vascular endothelial tissues (i.e., endothelia). Especially, the mechanical property of the triple-layer arterial vessels can well meet the anti-stress requirements as human blood vessels. In a word, a small-diameter arterial vessel was successfully constructed through the combined mold and has a promising application prospect as a clinical small-diameter vessel graft.

## 1. Introduction

The incidence of cardiovascular disease is high all over the world, and the number of deaths caused by cardiovascular disease each year exceeds the total of deaths from cancer [1]. The vasculature of the human body is composed of three distinct parts with complex branch structures, which aim to effectively provide oxygen and nutrition exchange for the whole-body cells and take away metabolic wastes in time [2]. Normally, oxygen-rich blood is delivered from heart to aorta (diameter > 6 mm), arteries (diameter > 1 mm), arterioles (diameter from 0.3 to 1 mm) and capillary (diameter between 10 and 15 µm) bed sequentially [3]. Arteries play an important role in supplying the oxygen-rich blood to the whole human body.

A typical artery of mammals can be roughly divided into triple layers from the inside to the outside. The innermost layer is termed tunica intima, in which endothelial cells (ECs) are arranged in sequence under the influence of shear stress of blood flow and act as a selective barrier of nutrients, hormones and ions, with anticoagulant function [2]. The middle layer, tunica elastic (or media), is composed of elastic fibers, collagen fibers and a large number of vascular smooth muscle cells (SMCs). SMCs function as vasoconstrictors and vasodilators, altering the mechanical strain on blood vessels to allow for the pulsatile flow of blood t in response to stress or injury. In angiogenesis, SMCs play a vital role in matrix secretion as well as the propagation of diseases such as atherosclerosis and hypertension [4]. With the beating of the heart, the middle layer of large arteries contract and relax accordingly. The outermost layer is called tunica adventitia, containing fibroblasts, or connective tissues, and nerve fibers [5]. Until present, no bioartificial arteries had been created with a similar composition, framework and function of their natural counterparts.

With the development of biomaterials, vessels with diameter larger than 6 mm can be replaced by unbiodegradable polymer, such as polytetrafluoroethylene, to repair aortal and femoral arteries. The initial clinical trial of living large-caliber vessels in a pediatric patient was reported in 2001 by Shin’oka and colleagues. Briefly, a 1 cm diameter biodegradable tube was seeded with autologous myofibroblasts and SMCs, cultured for 1 week in vitro, and transplanted as a replacement to an occluded pulmonary artery. The authors reported on high patency rates with no signs of graft occlusion 7 months post implantation [6]. Nevertheless, some terminal diseases such as coronary atherosclerosis or occlusion require smaller diameter blood vessel to replace the lesion [7]. At present, autologous vein or artery transplantation is often applied in small-diameter vascular bypass or replacement surgery [3]. This method often brings great pain to patients. In view of the increasing number of cardiovascular patients and the limited choice of autologous vascular transplantation, there is an urgent need to develop clinical alternatives [8].

There are two commonly used blood vessel creation methods in the fields of tissue engineering and organ manufacturing. One is rapid prototyping, also known as additive manufacturing [9], including cell-laden polymer assembly or three-dimensional (3D) bioprinting containing cells [10]. The other is combined-mold technology [11]. 3D bioprinting is defined as printing bioartificial tissues/organs with cell-laden hydrogels, such as extracellular matrix (ECM), in the form of ‘bioinks’ under the control of computer programs in a layer-by-layer fashion [12,13,14]. Since the beginning of the 21st century, 3D bioprinting has completely changed the field of biomedicine. The first cell-laden living 3D construct was printed by Professor Wang in 2003 [15]. At present, many kinds of vascular vessels can be constructed through 3D bioprinting and can be applied in clinical fields [16,17,18]. This kind of process, however, is strictly affected by cost, equipment and precision of formation. Accordingly, it is difficult to form tissues and organs with subtle structures. In addition, different printing equipment may have different negative effects on the activity of living cells.

Over the last two decades, Professor Wang has also exploited several series of combined-mold technologies besides the advanced multiple nozzle 3D bioprinting technologies for bioartificial organ manufacturing [19]. The combined-mold technologies arise at a historic moment. There are some similarities between the combined mold and 3D bioprinting technologies. For example, both of them need computer modeling, and both of them can establish sophistical models according to the target tissues/organs. An outstanding advantage of the combined-mold technologies is that cell density in the polymer solutions can be very high during the cell-laden polymeric material assembling stage. The commonly used mold-forging material is photopolymer resin, which is non-toxic to cells during the cell-laden polymer molding processes [20].

In this study, a small-diameter triple-layer artery was constructed in vitro through a combined-mold technology. There were three steps involved in the construction process. During the first step, adipose-derived stem cells (ASCs) were added into a gelatin/alginate solution containing endothelial growth factors (GFs) before being cultured for several days, simulating the inner endothelium layer (i.e., tunica intima) of natural arteries. During the next step, ASCs were loaded by the gelatin/alginate hydrogel containing smooth muscle GFs, mimicking the middle media (i.e., tunica elastic) of natural arteries. During the third step, synthetic polylactic-glycolic acid (PLGA) was applied on the bilayer bioartificial arteries, acting as the outer adventitia of natural blood vessels. Mechanical strength of the bioartificial arteries was increased sharply with the synthetic PLGA layer. After several days of in vitro cultures, cell states in the gelatin/alginate hydrogels were very well. Under microscope, the functional layers of the bioartificial arteries could be clearly observed and their mechanical performance could meet the requirements of in vivo transplantation.

## 2. Results

### 2.1. Biocompatibility of PLGA

**Figure 1:**
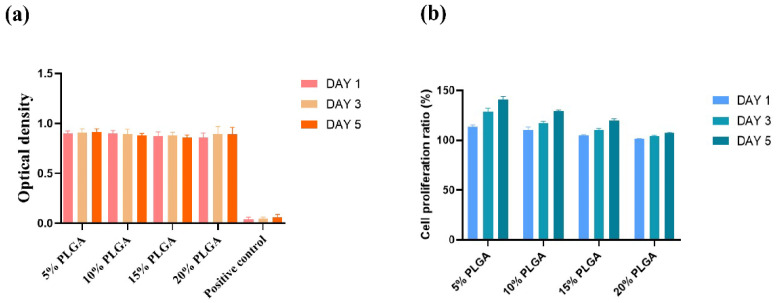
Biocompatibilities of PLGA with different concentrations: (**a**) cytotoxicity of PLGA with 5%, 10%, 15% and 20% concentrations (at 37 °C, *n* = 3); (**b**) cell proliferation ratios of PLGA with 5%, 10%, 15% and 20% concentrations (at 25 °C, *n* = 3).

Biocompatibilities of PLGA membranes with different concentrations were characterized through cytotoxicity (Figure 1a) and cell proliferation ratios (Figure 1b). The optical density (OD) values were all above 0.8 at three-time nodes (absorbance above 0.8 could be considered non-toxic to cells), and there was no significant difference between them. From a statistical point of view, PLGA membranes after PBS extraction can be considered to be non-toxic to cells. The higher the concentration of PLGA, however, the lower the cell proliferation ratio. The cell proliferation ratio on the fifth day of 20% PLGA solution was lower than that on the first day of 5% PLGA solution. Considering the results of the cell proliferation ratios, 5% and 10% PLGA solutions were selected for the subsequent experiments.

### 2.2. Mechanical Properties of PLGA

**Figure 2:**
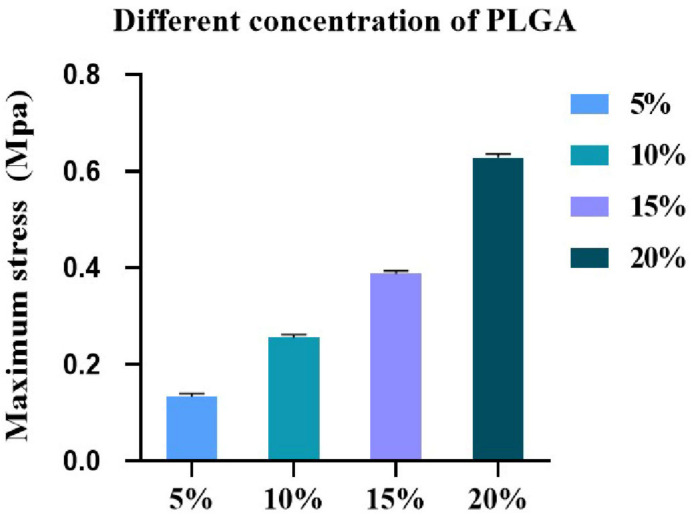
Maximum stress of the PLGA membranes with different concentrations (at 25 °C, *n* = 3).

The maximum stress performance of PLGA with different concentrations is shown in Figure 2. The maximum stress of 5%, 10%, 15% and 20% PLGA is 0.133 ± 0.01 Mpa, 0.252 ± 0.03 Mpa, 0.387 ± 0.04 Mpa and 0.623 ± 0.06 Mpa, respectively. It can be seen that the maximum stress of PLGA increases with the increase of its concentration.

The maximum elongation at fracture of different PLGA membranes with different concentrations is shown in Table 1. The concentration of PLGA is proportional to its maximum elongation at fracture but not linearly.

### 2.3. Preparation and Differentiation of ASCs

**Figure 3:**
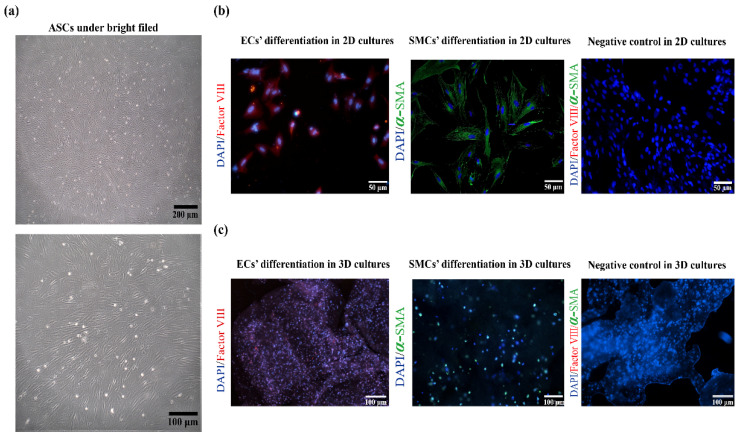
ASCs that were used for the arterial construction and induced to differentiate into endothelial cells (ECs) and smooth muscle cells (SMCs): (**a**) morphology of ASCs under optical microscope; (**b**) ASCs were induced into ECs and SMCs, respectively, after 5 days in vitro 2D cultures. Factor VΙΙΙ protein expressed red fluorescence, alpha-smooth muscle actin (α-SMA) expressed green fluorescence and nuclei expressed blue fluorescence; (**c**) ASCs were induced into ECs and SMCs, respectively, in the 3D gelatin/alginate hydrogel after 5 days of in vitro cultures with protein of Factor VΙΙΙ (red), α-SMA (green) and nuclei (blue) expression, respectively.

Adherent ASCs that are in a logarithmic growth cycle are shown in Figure 3a. It can be observed that the morphology of the ASCs is typical of mesenchymal stem cells. The states of the cells have a close relationship with the cell numbers, and some of the cells grow into a vortex shape in some visual fields. Figure 3b,c shows some ECs and SMCs differentiated from ASCs on 2D and 3D cultures. After 3 days’ treatment with ECs’ culture media and SMCs’ culture media, respectively, ECs protein factor VΙΙΙ (red) and SMCs protein alpha-smooth muscle actin (α-SMA, green) were expressed clearly (Figure 3b) through immunofluorescence staining. Similarly, after 5 days’ 3D culture, ECs’ protein factor VΙΙΙ and SMCs protein α-SMA were manifested in the hydrogels (Figure 3c). It can be confirmed that ASCs can be induced to differentiate into ECs and SMCs in the hydrogels through ECs’ and SMCs’ culture media, respectively, which lays the foundation for the in vitro construction of EC and SMC layers in the artificial arterial vessels.

**Figure 4:**
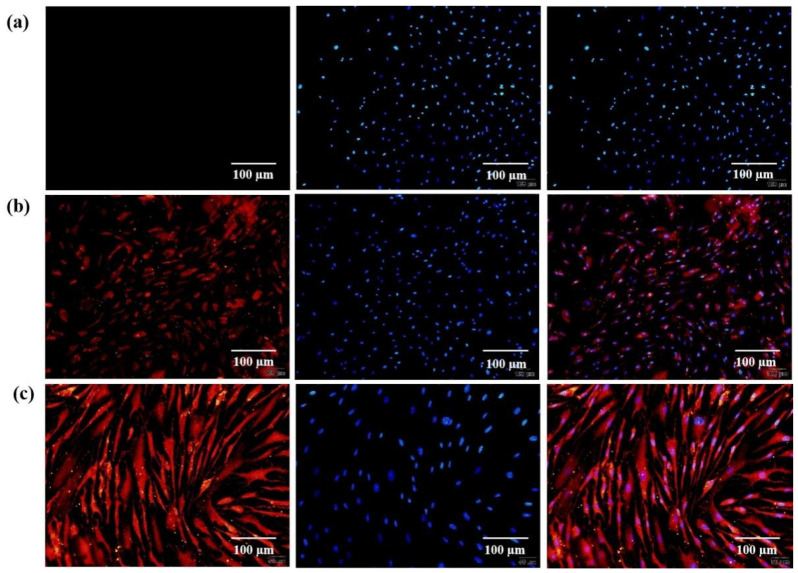
ASCs that were used for the arterial construction and induced to differentiate into endothelial cells (ECs) and smooth muscle cells (SMCs) after 14 days of in vitro cultures: (**a**) control ASCs that were not induced into ECs and SMCs. In the first picture (the left line), there were nearly no Factor VΙΙΙ (red) and α-SMA (red) expression. In the second picture (the middle line), there was only nuclei (blue) expression. In the third picture (the right line), the cell states from the combination of the first two pictures were clear; (**b**) ASCs were induced into SMCs. Alpha-smooth muscle actin (α-SMA) expressed red fluorescence (left), nuclei expressed blue fluorescence (middle), and the first two pictures were combined (right); (**c**) ASCs were induced into ECs with the expressions of Factor VΙΙΙ (red, left), nuclei (blue, middle), and the combination of Factor VΙΙΙ and nuclei (right).

Additionally, more controls of the ASCs that were used for the arterial construction and differentiation after 14 days of in vitro cultures are shown in Figure 4. For the control ASCs that were not induced into ECs and SMCs, there were nearly no Factor VΙΙΙ (red) and α-SMA (red) expressions even after 14 days of in vitro cultures (Figure 4a). For the ASCs that were induced into SMCs, alpha-smooth muscle actin (α-SMA) expressed red fluorescence and nuclei expressed blue fluorescence (Figure 4b). For the ASCs that were induced into ECs, Factor VΙΙΙ expressed red fluorescence and nuclei expressed blue fluorescence (Figure 4c).

### 2.4. Live/Dead Staining of Cells in the Hydrogel

**Figure 5:**
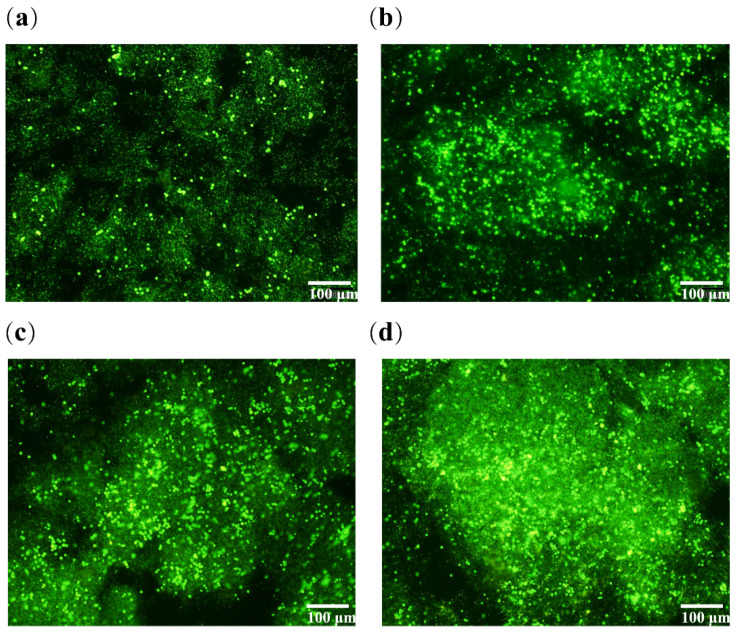
Diagram showing AO/PI cell-stained confocal images of ASCs in hydrogel cultured for different periods: (**a**) 1 day; (**b**) 3 days; (**c**) 5 days; (**d**) 10 days.

AO/PI staining of ASCs cultured in the hydrogel for 1 day, 3 days, 5 days and 10 days are shown in Figure 5, respectively. It can be witnessed that after being cultured in the hydrogel for 1 day, most of the ASCs are scattered and uniformly distributed in the hydrogel. There is no large scale of proliferation, and the cells are not closely related to each other. Thus, it is difficult to form dense tissues (Figure 5a). Cells’ density increases obviously after 3 days of in vitro culture, indicating that cells had adapted to the state of 3D cultures and proliferated. At this time point, connections between cells can be detected in the local visual field (Figure 5b). A further sharp increase in cell numbers was revealed after 5 days of in vitro culture, suggesting that cells in the hydrogel are in a logarithmic growth cycle and proliferate massively. Meanwhile, a large number of connections have formed between cells in some visual fields (Figure 5c). Cells are closely connected with each other after 10 days of culture. Large numbers of cells were stacked together in the visual field of Figure 5d, indicating that there is a tendency of regular aggregation or organization of the enhanced cells.

**Figure 6:**
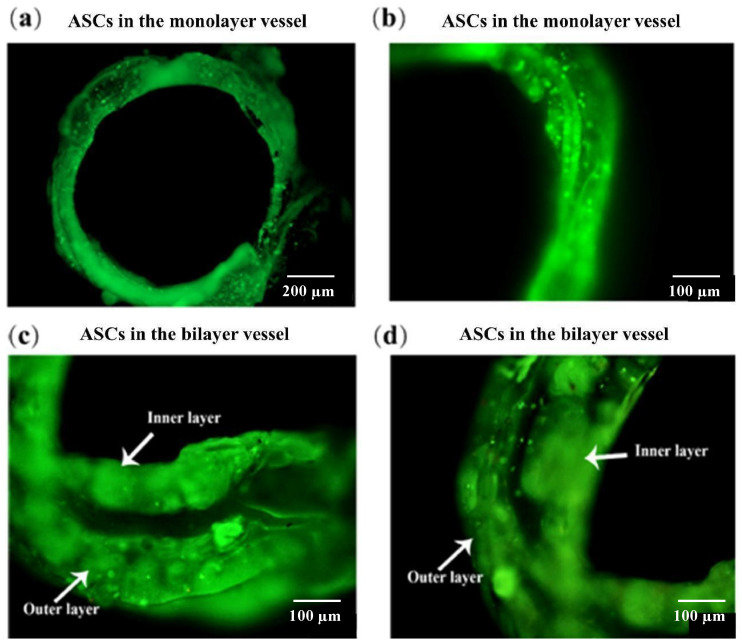
Diagram showing AO/PI cell-stained confocal images of ASCs in the vessel structures cultured for different periods: (**a**) ASCs cultured in monolayer vessel for 5 days; (**b**) enlarged view of monolayer vessel; (**c**) ASCs cultured in a bilayer vessel for 10 days; (**d**) ASCs cultured in another bilayer vessel for 10 days.

AO/PI staining results of the monolayer vessels after 5 days of culture are shown in Figure 6. It can be seen that after combined-mold construction, the monolayer blood vessels are circular, the structure is well maintained and ASCs are uniformly distributed in the vascular monolayer (Figure 6a). The distribution of ASCs in the monolayer of blood vessels can be further observed in Figure 6b. Individual cells grow into clusters and form connections between each other. There are no red dots (dead cells) in the visual fields, indicating that the ASCs have high adaptability to the hydrogel. Similarly, the live/dead staining of bilayer blood vessels cultured for 10 days are shown in Figure 6c,d. When the inner layers were cultured for 10 days, and the outer vessels were cultured for 5 days, the two layers of vessels were obviously detected but not stratified. Cells in each of the two layer are closely related to each other and grow into clusters. There are almost no dead cells, indicating that the cells in the double layer blood vessels also grow well and have high activities.

### 2.5. Morphology of the Arterial Vessels

**Figure 7:**
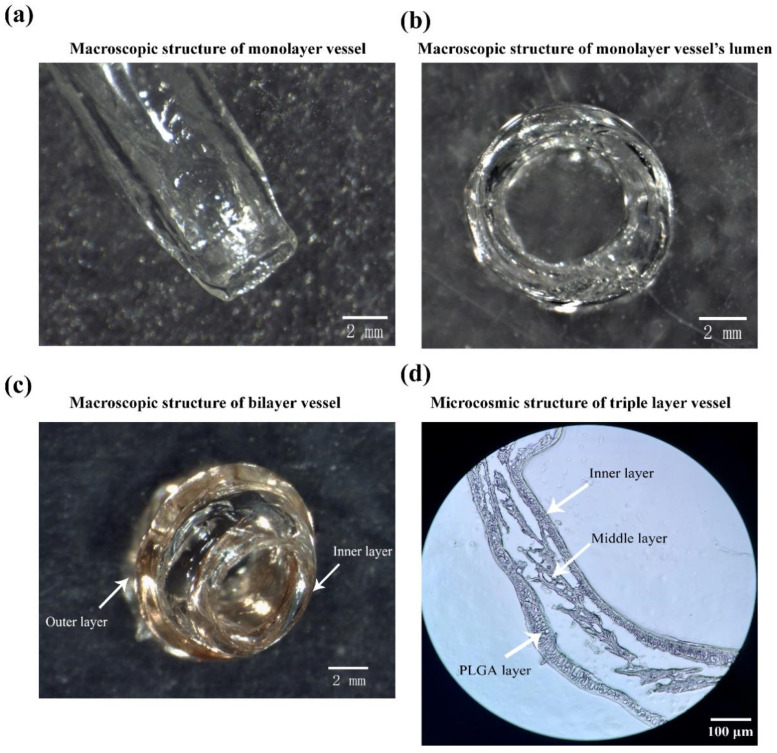
Structures of the constructed arterial vessels through the combined-mold techniques and solvent extractions (without cells): (**a**) an image of the constructed monolayer vessels under stereomicroscope; (**b**) a section of the monolayer blood vessels under stereomicroscope with clear annular and smooth lumen structures; (**c**) a diagram of the bilayer vessels under stereomicroscope; (**d**) a frozen section of the triple-layer arterial vessels with detailed microscopical structures.

Macroscopic structure of the constructed vessels without cells was characterized via stereomicroscope, as shown in Figure 7. The appearance of the constructed monolayer vessel is transparent, the vascular wall is smooth and the texture is uniform (Figure 7a). After trimming, the orifice of the monolayer vessels is even and smooth (Figure 7b). There is no messy structure around the monolayer vessel, indicating that the small-diameter blood vessel can be well shaped through the combined-mold technique. The color of the double layer vessels is not transparent after molding. It is difficult to characterize the blood vessels with bilayer structure. A small amount of eosin solution was added to the hydrogel before they were checked using a stereomicroscope, as shown in Figure 7c,d. The inner and outer structures of the bilayer vessels are obviously distinguished, and the internal surfaces are very smooth.

PLGA is tightly wrapped on the surface of the bilayer vessels after solvent extraction, and the extracted PLGA is a white coating. It is difficult to characterize the triple-layer vascular structures at the macro level. Accordingly, frozen sections are applied to characterize them at micro level, as shown in Figure 7d. The three layers with different components have been united as a whole and kept their shapes even after a series of solvent treatments, indicating that the triple-layer arterial vessels can be used as bioartificial arteries with living cells (the interconnected ECs and SMCs in the inner and middle layers), anticoagulation/permeation functions (the internal ASC-derived endoderm or endothelium), and anti-suture/anti-stress capabilities (the mechanical strong enough PLGA layer).

### 2.6. Immunofluorescence of the Vessel Structures

**Figure 8:**
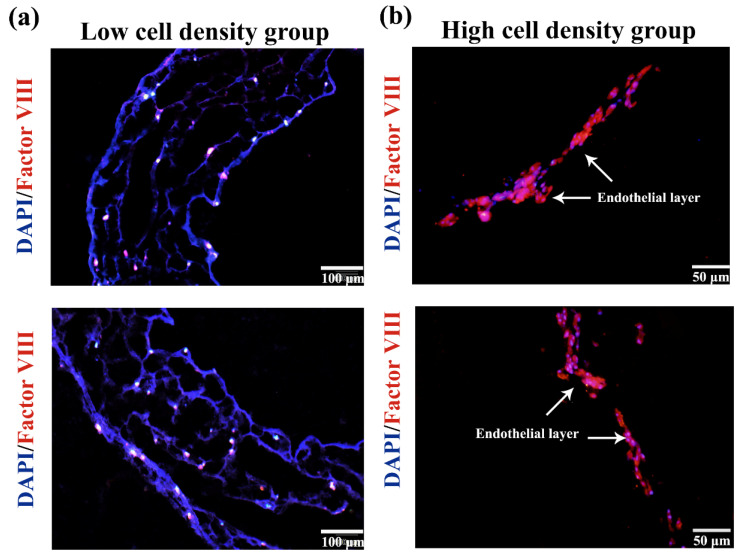
Immunofluorescence identification of the differentiated ASCs in the constructed vessels with frozen sections: (**a**) endothelial differentiation of the ASCs in the monolayer vessels with a low cell density; (**b**) endothelial differentiation of the ASCs in the monolayer vessels with a high cell density.

The immunofluorescence staining results of the monolayer vessels are shown in Figure 8. After the ASCs with a cell density of 1 × 10^6^ cells/mL were cultured in the gelatin/alginate hydrogel with endothelial growth factors for 5 days (Figure 8a), the cells attached to the hydrogel network and grew very well. ECs’ protein factor VΙΙΙ (red) had a positive expression, indicating that ASCs were completely induced into ECs. However, this cell density of ASCs before induction was not high enough, and there was no sign of sequence arrangement which is similar to a natural vessel’s endothelial tissue. When the density of ASCs was increased to 2 × 10^6^ cells/mL and cultured in the same hydrogel with endothelial growth factor for 10 days, the expression of ECs’ protein factor VΙΙΙ (red) had been greatly increased. Meanwhile, the neighbor cells were intimately related to each other with strong overlapped red immunofluorescence, suggesting that all the involved ASCs had differentiated into ECs and the interconnected ECs had formed anti-thrombotic endothelium (Figure 8b). Due to the high cell density, long culture time and shear stress produced by the change of culture medium, the differentiated ECs undergo the phenomenon of sequence arrangement, which makes the internal surfaces of the constructs similar to natural endothelial tissues.

**Figure 9:**
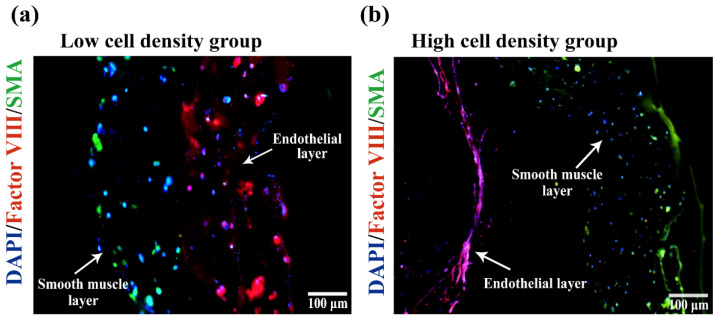
Immunofluorescence identification of differentiated ASCs in constructed vessels with frozen sectioins: (**a**) an image of the differentiated ASCs in the bilayer vessels with a low cell density; (**b**) a picture of the differentiated ASCs in the bilayer vessels with a high cell density.

In Figure 9a, ASC states, cultured in the bilayer arterial vessels, with a initial cell density of 1 × 10^6^ cells/mL, compounded with endothelial and smooth muscle growth factors for 10 days, are distinctly demonstrated in the frozen sections. Unlike those ASCs, with low initial cell density and short term growth factor induction (Figure 8a), all the ASCs had differentiated into ECs and SMCs in the respective double layers, which is consistent with the endothelium and medium structures of the natural arteries. There is a certain trend of sequential arrangement between cells under shear stress. Due to the low cell density, however, the relationship is not close enough to generate endothelial tissues. Most of the differentiated cells still scatter in the gelatin/alginate hydrogel with no obvious sign of new tissue formation. In contrast with the low cell density group, the differentiated cell states, with a initial cell density of 2 × 10^6^ cells/mL, are totally changed, as shown in Figure 9b. Especially on the surface of the endothelium layer, nearly all the ECs had spread out with wide and long pseudopods, like the cells extremely in the natural blood vessels. There are intimate relationships among the differentiated ECs in the internal surfaces of the bioartificial arteries. The phenomenon of sequential arrangement of cells, which makes the construct similar to the natural vascular endothelial tissues, is a fundmental requirement for blood vessel manufacturing and functionization. Due to the short culture time and low effect of shear stress on the double layer artificial arteries, most of the induced SMCs still distribute randomly in the arterial structures of the outer layer. Few of them connect with each other producing sequential arrangement.

### 2.7. HE Staining of the Arterial Vessels

**Figure 10:**
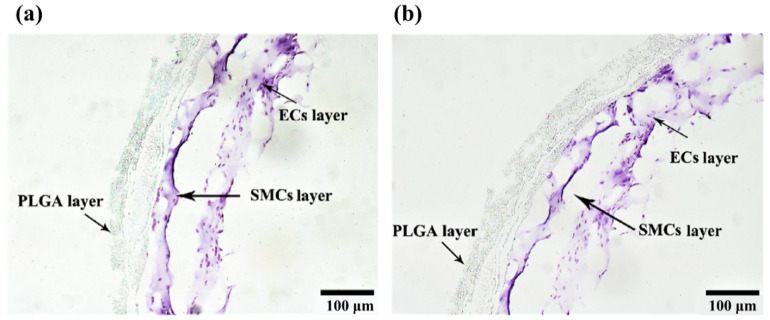
HE staining results of the triple arterial vessels with paraffin sections: (**a**) a triple-layer arterial vessel structure under bright filed; (**b**) another triple-layer arterial vessel structure under bright filed.

HE staining results of the ASCs cultured in the triple-layer arterial vessels for 10 days are shown in Figure 10. There are three distinctive layers with different components, comprising two layers of cell-laden natural hydrogels and one layer of synthetic polymer PLGA. The three different layers link (i.e., connect) each other closely withou delamination. Cell states in the hydrogels and stained in purple are very clear, a dominant phenomenon in natural arterial tissues. The inner layer and middle layer structures can be differed from the macro pores generated from the disappeared hydrogels. Most of the differentiated cells no longer exist in a single or cluster shapes but in a uniform tissue states. However, because the culture time of cells in the middle layer is relatively shorter than those in the inner layer, the oriental arrangement trend is not as obvious as that in the internal surface of the inner layer. In general, the vascular structure is smooth and uniform as a whole, and the stratification can be obviously witnessed.

### 2.8. Mechanical Properties of the Bioartificial Arteries

**Figure 11:**
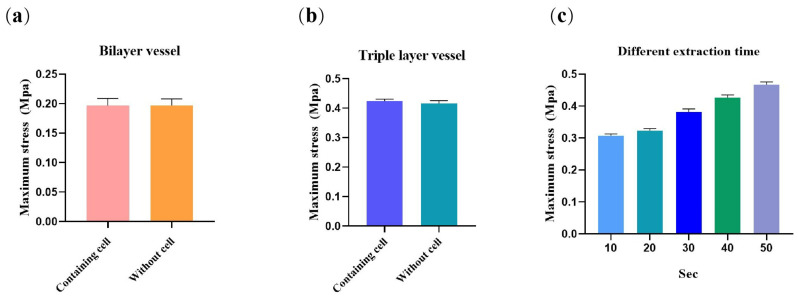
Mechanical properties of constructed bioartificial arteries: (**a**) maximum stress of the bilayer vessels (at 25 °C, *n* = 3); (**b**) maximum stress of triple-layer vessels (at 25 °C, *n* = 3); (**c**) maximum stress of the triple-layer vessels with different PBS extraction time (at 25 °C, *n* = 3).

Mechanical properties of the bioartificial arteries were characterized from three aspects: bilayer vessels, triple-layer vessels and PLGA membranes with different extraction times. The results are shown in Figure 11. Maximum stress of the bilayer blood vessels with or without cells are 0.191 ± 0.04 Mpa and 0.188 ± 0.07 Mpa, respectively (Figure 11a). Maximum stress of the triple-layer blood vessels with or without cells are 0.421 ± 0.12 Mpa and 0.416 ± 0.10 Mpa, respectively (Figure 11b). It can be concluded that the contribution of the polymer materials to the mechanical properties of the blood vessels is major, and the contribution of cells to the mechanical properties is relatively minor. From the data, it can be seen that there is no significant difference between the cell-containing group and the non-cell-containing group. Mechanical properties of triple-layer blood vessels after different PBS extraction time, i.e., 20 s, 30 s, 40 s, 50 s and 60 s, are 0.307 ± 0.03 Mpa, 0.331 ± 0.04 Mpa, 0.376 ± 0.02 Mpa, 0.422 ± 0.04 Mpa, and 0.468 ± 0.07 Mpa, respectively (Figure 11c).

Maximum deformations of the bilayer and triple-layer vessels are shown in Table 2, while the effects of different extraction times on the deformation of the triple-layer constructs is shown in Table 3.

## 3. Discussion

Normally, natural polymers have good biocompatibilities and poor mechanical properties, while synthetic polymers have super mechanical properties and inertial/unideal biocompatibilities. The combination of natural and synthetic polymers is a feasible way to manufacture bioartificial organs with both of their merits, such as anti-suture and cell growth capabilities [21,22,23,24,25,26].

During the bioartificial artery construction processes, natural polymers, gelatin and alginate are dissolved in inorganic solvent, such as water or PBS, which can be used to assemble cells directly, while synthetic PLGA needs to dissolve in organic tetraglycol, which has some toxic effects when the tetraglycol molecules contact cells. Thus, the tetraglycol molecules need to be extracted from the PLGA layer after the artery construction stage. Figure 1a shows that the PLGA layer after extraction was almost non-toxic to cells. The activities of ASCs have negative relationships with the PLGA concentrations. Figure 1b indicates that the higher the concentration of the PLGA solution, the less favorable it is to cell proliferation. This is because the higher the concentration of the polymer solution, the smaller the pore size of the PLGA membrance. Consequently, it is more difficult to provide nutrients to cells through the culture medium.

Hydrogels with different components may demonstrate different colors [27,28,29]. The pure gelatin/alginate hydrogel is transparent after CaCl_2_ crosslinking, which can be clearly characterized under the microscope. Nevertheless, it is difficult to characterize the bilayer arterial structures under stereo microscope due to the influence of light refraction. Ideally, different colors can be applied to distinguish the different layers of the bioartificial arteries. Because the eosin dye solution is water-soluble, it does not change the hydrogel property obviously. Accordingly, we added some eosin into the hydrogel to construct the bilayer vascular structures, as Figure 7c shows. The triple-layer vascular structures, containing synthetic polymer PLGA, are hard to be characterized using optical microscopes. Thus, we use the frozen sections to characterize the micro structures of the triple-layer bioartificial arteries, just as manifested in Figure 7d.

After compounding different growth factors with hydrogels containing ASCs, the arterial vessels with triple layers were successfully constructed via the combined-mold technology. The triple layers are the inner layer (EC layer), the middle layer (SMC layer) and the outer layer (PLGA). Initially, in order to verify that the induction solution of such formula can induce ASCs to differentiate into ECs and SMCs, respectively, the identification experiments were carried out on the 2D cultures. Results showed that the induced differentiation solutions could promote the differentiation of ASCs into ECs and SMCs rapidly (3 days). Meanwhile, ASCs can also be induced to differentiate into ECs and SMCs quickly (5 days) in the hydrogels (containing growth factors), which lay the foundation for the subsequent vascularization experiments (Figure 8, Figure 9 and Figure 10).

ASCs grew well in the hydrogel after being cultured in vitro for 10 days. Cell proliferation rate inside was fairly high. Compared with the 2D cultures, the 3D structures of the cell-laden hydrogels can better simulate cell’s growth environments in vivo. When the ASCs were embedded in the hydrogel containing EC growth factors, they were easily induced to differentiate into ECs after several days’ in vitro culture, resulting in the formation of the inner endothelial layer. The model of endothelial blood vessel was obtained. The shear stress caused by blood flow in blood vessels can promote angiogenesis and the sequential arrangement of the endothelial cells, enhance the interconnection between cells and increase the barrier function of endothelial cells and vascular maturation [30,31]. After being cultured in vitro, the culture medium was used to wash the blood vessels to simulate the shear stress caused by blood flow. Immunofluorescence results of vascular sections showed that under the influence of shear stress, there was a positive relationship between the cell connections and phenomenon of sequence arrangements when the cell density was high enough, which is similar to the trend of natural endothelial tissues.

After 5 days of culture, the endothelia vascular inner layer structure was molded into a layer of ASCs with smooth muscle growth factor containing hydrogel on its outer layer through the combined mold technology. The vascular structure of the middle layer was formed. Immunofluorescence results of the vascular section suggested that the ASCs in the middle layer were successfully induced to differentiate into the SMC layer. It is very interesting that, in the high cell density group, the differentiated SMCs were also related to each other closely due to the influence of shear stress, and the phenomenon of sequence arrangement appeared, which is similar to the natural tunicae media. After the bilayer structures were immersed in PLGA solutions, covered by PLGA membrances, and the solvent tetraglycol molecules were extracted from the PLGA membrances using PBS, the triple-layer arterial vessels remained very well. Results of HE staining showed that the vascular structure of the triple layers of arterial vessel was clear and the cell states were obvious (Figure 10). No any adverse effects on the living cells were found with the coating and extraction procedures.

Normally, the blood pressure in the human body is between 100–120 mmHg (13.33–16 Kpa, systolic blood pressure) and 60–80 mmHg (8–10.67 Kpa, diastolic blood pressure). Through the mechanical test of the triple-layer blood vessels, it is found that the maximum radial strain is 0.421 ± 0.04 Mpa and maximum strain is 1.499 ± 0.04%, which can meet the requirements for being used as small-diameter arteries. In the natural artery, the mechanical property is mainly determined by the SMCs and elastic fibers in the middle layer. In this study, the mechanical properties of the bioartificial arteries were mainly lying on the outer-layer synthetic polymer PLGA; meanwhile, the contribution of the newly formed SMCs in the middle layer can be ignored. This is very important for the bioartificial arteries to be implanted in the human body with anti-suture capabilities.

## 4. Materials and Methods

### 4.1. Preparation of Hydrogel

Gelatin (glue strength ~250 g Bloom, CSA: 9000-70-8) was purchased from Aladdin (Shanghai Aladdin Biochemical Technology Co., Ltd., Shanghai, China). Sodium alginate (CAS: 9005-38-3) was purchased from Macklin (Shanghai Macklin Biochemical Co., Ltd., Shanghai, China). Transglutaminase (TG) (120 U/g, CAS: 80146-85-6) was purchased from Aladdin. CaCl_2_ (CAS: 10043-52-4) was purchased from Macklin. PLGA (LA/GA = 70/30, Mw = 100,000 DA) was purchased from Jinan Daisheng Biotechnology Co., Ltd., (Shandong, China). Other reagents used in the experiments were all of analytical grade.

In our previous study, we found that the cell proliferation ratio is relatively high in the hydrogel composed of 4% (*w*/*v*%) gelatin and 1.5% (*w*/*v*%) sodium alginate [19]. Accordingly, the hydrogel used in this study also contained 4% gelatin and 1.5% alginate. Gelatin and alginate were weighted, respectively, before being dissolved in deionized water and heated in a water bath at 75 °C for 1 h.

### 4.2. Preparation of ASCs

ASCs were isolated from Sprague-Dawley (SD) rats (120–140 g). Briefly, after the inguinal adipose tissues were isolated, washed with sterilized phosphate buffer (PBS) and cut into pieces, they were digested with collagenase Ι (1 mg/mL) (Solaibio Life Science, Beijing, China) and cultured with Dulbecco’s modified Eagle’s medium/F12 (DMEM/F12, Corning Incorporated, Corning, NY, USA) containing 15% heat-inactivated fetal bovine serum (FBS, LONSERA, Shanghai Shuangru Biology Science & Technology Co., Ltd.) and 1% penicillin/streptomycin (NCM Biotech, Suzhou, China) at 37 °C, 5% CO_2_. Cells at passage 3–4 were used for the subsequent experiments.

### 4.3. Biocompatibility of PLGA Membranes

Cytotoxicity and cell proliferation ratios were detected using CCK-8 Cell Counting Kit (Bioss Antibodies, Beijing, China) according to the instructions. For cytotoxicity tests, three groups of experiments were set up: experimental group, positive control group (75% ethanol) and blank control group. Briefly, PLGA was dissolved in tetraglycol (Macklin, Shanghai Macklin Biochemical Co., Ltd., Shanghai, China) at 60 °C to prepare 5%, 10%, 15% and 20% PLGA solutions. The prepared PLGA membranes with four concentrations were extracted with PBS, freeze-dried and sterilized, respectively. Afterward, the samples of each group were soaked in a DMEM/F12 medium of 4 mL (15% fetal bovine serum, 1% penicillin-streptomycin) and incubated for 24 h to get the supernates. Then, ASCs were added into a 96-well culture plate at a density of 5 × 10^3^ cells/mL. After the cells adhered to the culture plates, the leached liquor mixed with CCK-8 (leach liquor: CCK-8 = 9:1) was added on days 1, 3 and 5 and incubated at 37 °C for 60 min. Then, optical density (OD) was recorded using a microplate reader (Multiskan FC, Thermo Scientific, LOGAN, UT, USA) at a wavelength of 450 nm. The light absorption value of the experimental group, positive control group and blank control group was denoted as *Ode*, *Odc* and *Odn*, respectively. Cytotoxicity was calculated using the following equation:

(1) OD=(ODe−ODn)/(ODc−ODn) 

The method for cell proliferation ratio tests is similar to that of the cytotoxicity tests. Briefly, three groups were set up: experimental group (hydrogel with cells and PLGA film), control group (hydrogel with cells only) and blank control group (hydrogel without cells). After the ASCs were added into the gelatin/alginate solutions with a density of 5 × 10^3^ cells/mL, 100 µL of the cell-laden solution was moved to a well of a 96-well plate and crosslinked for 10 min using 20 µL of CaCl_2_ solution. An amount of 20 μL of 5%, 10%, 15% and 20% PLGA solutions were added to the surface of the cell-laden hydrogels, respectively. Then, 100 μL of sterile PBS solution was added to the surface of the PLGA membrance (or film) and cultured at 5% CO_2_, 37 °C. After 1, 3 and 5 days of in vitro cultures, the culture media was removed. Then, 100 μL of culture media containing CCK-8 (CCK-8: culture solution = 1:9) was slowly added and incubated for about 60 min. Subsequently, the supernatant of each well was sucked and transferred to a new 96-well plate; *OD* was recorded using the same microplate reader. The light absorption value of the experimental group, control group and blank control group was denoted as *ODe*, *ODc* and *Odn*, respectively. The cell proliferation ratio was calculated using the following equation:

(2) CP=(ODe−ODn)/(ODc−ODn)×100%

### 4.4. Mechanical Property of the PLGA Membrane

Four concentrations of PLGA solutions were added in an amount of 200 μL to a 24-well plate, which was evenly spread on the bottom of the well. After extraction, they were prepared into round sheets before soaking in PBS for 5 min. The samples remained moist during the tests and were placed between the upper and lower ends on the retainer of a microcomputer-controlled biomechanical testing machine (Qixiang testing instrument Co., Ltd., Shanghai, China). The stretch speed was set up to 5 mm/min. The maximum stress and strain were recorded after the samples were completely broken.

### 4.5. Arterial Angioplasty Design

**Figure 12:**
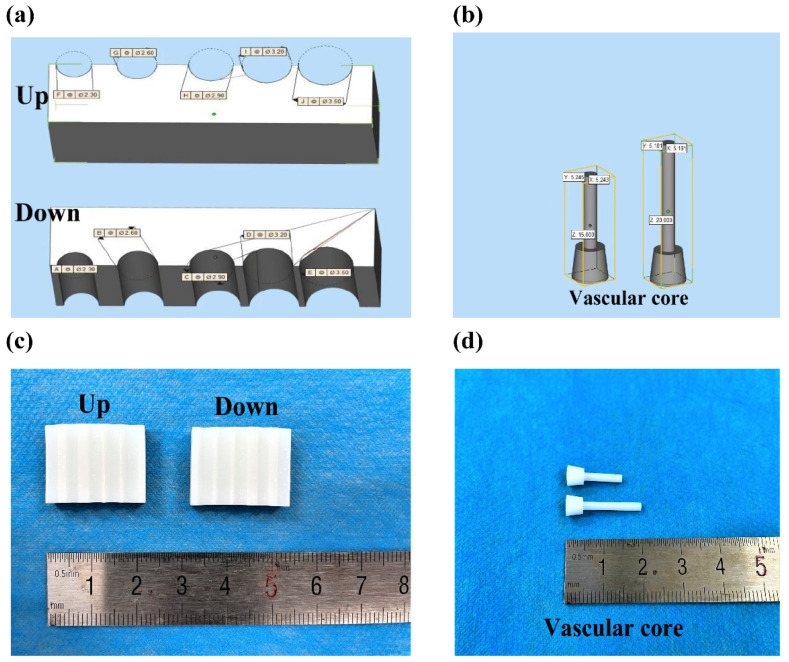
Diagram of the artery formation combined molds: (**a**) design of the upper and lower molds; (**b**) blueprint of the vascular cores; (**c**) the 3D-printed combined molds using photosensitive resin as raw material; (**d**) the 3D-printed vascular cores using photosensitive resin as raw material.

Figure 12 shows the schematic design of the artery formation combined molds. In Figure 12a, the combined molds include two parts: the upper mold and the lower (or down) mold. In Figure 12b, there are two vascular cores. The length of the vascular core is 10–20 mm and the diameter of the blood vessel is 3–6 mm, which can meet the preparation requirements of arteries with different lengths and inner diameters. The true molds were obtained through 3D printing, as shown in Figure 12c,d.

### 4.6. Construction of the Arterial Vessels

**Figure 13:**
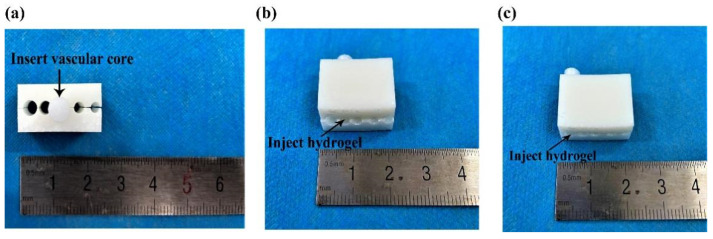
Schematic illustration of the arterial vessel construction processes using the combined molds: (**a**) after the upper and lower molds were combined, a series of go-through holes with different diameters were created between the molds, and a vascular core was inserted into one of the holes with a similar diameter with the core; (**b**) after the cell-laden gelatin/alginate solution was injected into the lumen between the core and go-through hole, the alginate polymers were crosslinked to form the first layer of cell-laden hydrogel; (**c**) after the cell-laden gelatin/alginate solution was injected into another lumen between the core and go-through hole, the alginate polymers were crosslinked to form the second layer of cell-laden hydrogel.

Figure 13 shows the angioplasty process. Briefly, the whole mold was immersed in 75% ethanol, disinfected for 15 min and dried naturally in a super-clean bench. After the upper and lower molds were combined, a series of holes with different diameters were created between the upper and lower molds. Then, the vascular core was inserted into a hole with a smaller inner diameter (Figure 13a), and the cell-laden gelatin/alginate solution with endothelial growth factors was injected into the lumen between the vascular core and combined molds from another end (Figure 13b). The whole mold system was then immersed in the crosslinking CaCl_2_ solution for 10 min to form the monolayer (or inner) vascular structure. Afterward, the vascular core together with the cell-laden hydrogel was pulled out from the first hole and inserted into another hole with a larger inner diameter, the cell-laden gelatin/alginate solution with SMCs’ growth factors was injected to the lumen through another end, and CaCl_2_ crosslinked for 10 min to obtain a bilayer vascular construct (Figure 13c). A similar covering process was repeated with PLGA solution to form the third layer of the bioartificial arteries. The tetraglycol solvent in the PLGA layer was finally extracted with PBS.

For the construction of the triple layer arteries, ASCs’ and ECs’ GFs were added into the gelatin/alginate solution at a density of 1 × 10^6^ cells/mL (low density group) or 2 × 10^6^ cells/mL (high density group) before they were injected into the holes of the combined molds. After the monolayer, or the inner layer, of the arterial vessel was cultured for 5 days with the vascular core, and the second layer of ASC-laden gelatin/alginate solution containing SMCs’ GFs was applied on the surface of the inner layer with a cell density 1 × 10^6^ cells/mL (low density group) or 2 × 10^6^ cells/mL (high density group). The vascular core was removed from the artificial arteries after the third PLGA was enwrapped. After the organic solvent tetraglycol was extracted thoroughly from the PLGA layer, the triple-layer arteries were cultured at 5% CO_2_, 37 °C, and the culture media was changed every day.

### 4.7. Differentiation of the ASCs and Identification

Growth factor is a kind of small molecular substance that can bind to different cell receptors, activate downstream signal pathways and cause a series of responses, which can stimulate cell growth, proliferation and differentiation. Different GF combinations were utilized to induce the ASCs to differentiate into ECs and SMCs. In the human body, ECM is the site where a large number of GFs are stored. Under the stimulation of some physiological or pathological factors, GFs in the ECM are selectively released. In this study, the GFs for the ECs are vascular endothelial growth factor (VEGF) and basic fibroblast growth factor (b-FGF) [11,20], while PDGF-BB, platelet derived growth factor (PDGF) and transforming growth factor β (TGF-β) were used for the SMC generation [20]. The formula of endothelial GFs and SMCs are shown in Table 4 and Table 5, respectively.

The identification of the ASC differentiated ECs and SMCs were confirmed via immunofluorescence. Briefly, after 3 days’ culture, the cells were fixed with 4% paraformaldehyde and permeabilized with 0.5% Triton X-100 solution. Then, the samples were blocked with 5% bovine serum albumin (Solaibio Life Science, Beijing, China) for 30 min. Afterward, the samples were incubated with rabbit anti-factor VΙΙΙ primary antibody (1:200, Biosynthesis Biotechnology Inc, Beijing, China) or mouse anti-smooth muscle actin primary antibody (1:600, Biosynthesis Biotechnology Inc, Beijing, China) overnight at 4 °C before they were sequentially incubated with goat anti-rabbit secondary antibody labeled with Alexa Fluor 555 (1:200, Biosynthesis Biotechnology Inc, Beijing, China) or goat anti-mouse secondary antibody labeled with FITC (1:200, Biosynthesis Biotechnology Inc, Beijing, China) for 1 h. To stain the nuclei, 4′,6-diamidino-2-phenylindole (DAPI, Solaibio Life Science, Beijing, China) was used. The images were observed under an Olympus TH4-200 confocal microscope (Olympus Corporation, Tokyo, Japan).

### 4.8. Live/Dead Cell Staining

Acridine orange (AO) is a kind of fluorescent dye with cell permeability, which was first extracted from coal tar at the end of the 19th century and has been widely used in the textile industry and as an antimicrobial agent [24]. Under the fluorescence microscope, AO can bind to DNA and RNA in the nucleus through the cell membrane, making the cells show green or yellowish green fluorescence. Propidium iodide (PI) is a DNA-binding dye that has no cellular permeability and can only stain dead cells. Under the fluorescence microscope, the normal cells could not be stained and the apoptotic cells were red. In order to clearly identify the live and dead states of the adipose stem cells in the vascular structures, an AO/PI double-staining cell-apoptosis detection kit was used.

For the AO/PI double-dye kit (BestBio, Shanghai, China) staining, 1 mL of the cell-laden polymer solutions with a density of 1 × 10^5^ cells/mL was added to a 24-well plate and crosslinked for 10 min using 1 mL of the CaCl_2_ solution. After crosslinking, 1 mL culture medium was added for cell cultures. The culture medium was changed every 2 days. After 1, 3, 5 and 10 days of the 2D in vitro cultures, 5 and 10 days for the 3D bilayer vessel cultures, a part of the cell-laden hydrogels was taken out, replaced into a 5 mL EP tube and cleaned thoroughly with PBS. The pre-prepared AO/PI solution was added to immerse the samples before they were incubated in the dark at 4 °C for 30 min and observed under an Olympus TH4-200 confocal microscope (Olympus Corporation, Tokyo, Japan).

### 4.9. Morphological Analysis of the Arterial Vessels

In order to verify that ASCs in the monolayer vessel were successfully differentiated into ECs, frozen section immunofluorescence was carried out on the 5 days’ culture samples. Briefly, after frozen section, the samples were blocked in 5% bovine serum albumin (Solaibio Life Science, Beijing, China) for 30 min and incubated with rabbit anti-factor VΙΙΙ primary antibody (1:200, Bioss Antibodies, Beijing, China) overnight at 4 °C. Then, the samples were incubated with goat anti-rabbit secondary antibody labeled with Alexa Fluor 555 (1:200, Bioss Antibodies, Beijing, China) for 1 h before being stained with DAPI (Solaibio Life Science, Beijing, China) for nuclei and observed under an Olympus TH4-200 confocal microscope (Olympus corporation, Tokyo, Japan).

As for verification of the bilayer vessels, double immunofluorescence staining of sections was applied. Similarly, the samples were blocked in 5% bovine serum albumin (Solaibio Life Science, Beijing, China) for 30 min after frozen section and incubated with rabbit anti-factor VΙΙΙ primary antibody (1:200, Bioss Antibodies, Beijing, China) and mouse anti-smooth muscle actin primary antibody (1:600, Bioss Antibodies, Beijing, China) overnight at 4 °C. After washing with PBS trice, the samples were incubated with goat anti-rabbit secondary antibody labeled with Alexa Fluor 555 (1:200, Bioss Antibodies, Beijing, China) and goat anti-mouse secondary antibody labeled with FITC (1:200, Bioss Antibodies, Beijing, China) for 1 h at 37 °C before being stained with DAPI (Solaibio Life Science, Beijing, China) for nuclei and observed under an Olympus TH4-200 confocal microscope (Olympus corporation, Tokyo, Japan).

### 4.10. Hematoxylin-Eosin (HE) Staining of the Arterial Vessels

Hematoxylin-eosin (HE) staining is the most basic and commonly used staining method in routine histological areas. In this study, paraffin sections of the bioartificial arteries were stained with HE. After dewaxing, the samples were stained with an improved HE staining kit (Solaibio Life Science, Beijing, China) according to the instructions.

### 4.11. Mechanical Property of the Arterial Vessels

Mechanical property is an important parameter for the actual performance of the bioartificial arteries. In order to explore whether the constructed small-diameter blood vessels can meet the clinical needs, a series of mechanical properties tests were carried out. Initially, some of the bilayer arteries without PLGA layer were tested to explore the contribution of cells to the vessel’s mechanics. The samples were trimmed to 5 mm long, and their two ends were fixed on the retainer of the mechanical testing machine. The tensile rate was set to 5 mm/min. Four samples were tested in each group. The triple-layer artery’s mechanics were carried out to mainly explore the contribution of PLGA to the vessel’s mechanical property. The extraction time was set up to 10, 20, 30, 40, 50 and 60 s, respectively, to explore the effect of different extraction degrees of PLGA to the artery’s mechanics.

### 4.12. Statistical Analysis

All the data were analyzed by GraphPad 8.0.2 version, which was created by Dr. Harvey Motulsky, The University of California, San Diego, American. A *p* value < 0.05 was considered to be statistically significant. Results were presented as the mean ± standard deviation (SD).

## 5. Conclusions

Based on a combined-mold technology, a small-diameter bioartificial artery with three-layer walls was successfully constructed in vitro. The inner and middle layers were constructed using ASC-laden gelatin/alginate hydrogels. Meanwhile, the outer layer was built via synthetic PLGA. To mimic the morphology of the natural artery, ASCs were mixed into gelatin/alginate hydrogels containing different GFs and cultured layer by layer before they were induced to differentiate into ECs and SMCs. When the cell density was high and the culture time was long enough, the arrangement of the differentiated ECs and SMCs in the hydrogels could be adjusted, simulating the natural endothelial and smooth muscle tissues with the influence of shear stress. The mechanical strength of the synthetic PLGA was strong enough to support the artificial arteries with anti-suture properties. This research provided a new way for the clinical applications of small-diameter blood vessels.

## Figures and Tables

**Table 1:** Maximum elongation fracture of PLGA membranes with different concentrations.

Concentration (%)	Maximum Elongation Fracture (%)
5	0.55 ± 0.09
10	1.38 ± 0.07
15	2.59 ± 0.08
20	3.21 ± 0.11

**Table 2:** Maximum deformation of the bioartificial arteries.

	Bilayer Vessels (%)	Triple-Layer Vessels (%)
Containing cells	0.398 ± 0.12	1.597 ± 0.13
Without cells	0.326 ± 0.05	1.499 ± 0.08

**Table 3:** Effect of different extraction times on deformation of the triple-layer arteries.

Extraction Time (s)	20	30	40	50	60
Maximum deformation (%)	0.371 ± 0.07	0.514 ± 0.08	0.778 ± 0.1	1.42 ± 0.13	1.47 ± 0.12

**Table 4:** Formula of ECs’ GFs.

GFs	Concentration
VEGF	50 ng/mL
b-FGF	10 ng/mL

**Table 5:** Formula of SMCs’ GFs.

GFs	Concentration
PDGF-BB	50 ng/mL
TGF- β1	10 ng/mL
b-FGF	2.5 ng/mL

## Data Availability

All the data are reliable and published online.
